# Machine learning approach for differentiating cytomegalovirus esophagitis from herpes simplex virus esophagitis

**DOI:** 10.1038/s41598-020-78556-z

**Published:** 2021-02-11

**Authors:** Jung Su Lee, Jihye Yun, Sungwon Ham, Hyunjung Park, Hyunsu Lee, Jeongseok Kim, Jeong-Sik Byeon, Hwoon-Yong Jung, Namkug Kim, Do Hoon Kim

**Affiliations:** 1 Department of Gastroenterology, Asan Medical Center, University of Ulsan College of Medicine, 88, Olympic-Ro 43-Gil, Songpa-gu, Seoul, 05505 Republic of Korea; 2 Department of Gastroenterology, Ilsan Paik Hospital, Inje University College of Medicine, Goyang, Republic of Korea; 3 Department of Radiology, Asan Medical Center, University of Ulsan College of Medicine, Seoul, Republic of Korea; 4 Department of Convergence Medicine, Asan Medical Center, Asan Medical Institute of Convergence Science and Technology, University of Ulsan College of Medicine, 88, Olympic-Ro 43-Gil, Songpa-gu, Seoul, 05505 Republic of Korea; 5 Department of Anatomy, Keimyung University School of Medicine, Daegu, Republic of Korea; 6 Department of Internal Medicine, Keimyung University School of Medicine, Daegu, Republic of Korea

**Keywords:** Gastroenterology, Engineering, Mathematics and computing

## Abstract

The endoscopic features between herpes simplex virus (HSV) and cytomegalovirus (CMV) esophagitis overlap significantly, and hence the differential diagnosis between HSV and CMV esophagitis is sometimes difficult. Therefore, we developed a machine-learning-based classifier to discriminate between CMV and HSV esophagitis. We analyzed 87 patients with HSV esophagitis and 63 patients with CMV esophagitis and developed a machine-learning-based artificial intelligence (AI) system using a total of 666 endoscopic images with HSV esophagitis and 416 endoscopic images with CMV esophagitis. In the five repeated five-fold cross-validations based on the hue–saturation–brightness color model, logistic regression with a least absolute shrinkage and selection operation showed the best performance (sensitivity, specificity, positive predictive value, negative predictive value, accuracy, and area under the receiver operating characteristic curve: 100%, 100%, 100%, 100%, 100%, and 1.0, respectively). Previous history of transplantation was included in classifiers as a clinical factor; the lower the performance of these classifiers, the greater the effect of including this clinical factor. Our machine-learning-based AI system for differential diagnosis between HSV and CMV esophagitis showed high accuracy, which could help clinicians with diagnoses.

## Introduction

Viral esophagitis is most commonly caused by herpes simplex virus (HSV) and cytomegalovirus (CMV) in immunocompromised patients and occasionally in immunocompetent patients^1^. The diagnosis of viral esophagitis is based on clinical history, endoscopic features, and histopathologic features. Clinically, the most common symptoms of HSV and CMV esophagitis are odynophagia, dysphagia, and chest pain^2,3^. The most important risk factor of HSV and CMV esophagitis is an immunocompromised status, including human immunodeficiency virus infection, organ transplantation, or malignancies^4,5^. Histopathology with specific immunohistochemical stains (IHC) or deoxyribonucleic acid (DNA) polymerase chain reaction (PCR) using tissues are required for definitive diagnosis of HSV and CMV esophagitis^4,6^. However, since tissue-based diagnostic evaluation takes several days for a result, immunocompromised patients with poor general conditions that require rapid treatment after rapid diagnosis often undergo empirical treatment before histological diagnosis.

When considering empirical antiviral agents, particularly in immunocompromised patients, endoscopic features are important for differentiating between HSV and CMV esophagitis until a specific diagnosis is made. According to several studies, the endoscopic features of HSV esophagitis include typically multiple, small, discrete, shallow ulcers with bullae or vesicles; yellowish exudate; and coalescence. The involvement of the middle to distal esophagus is most common^3,7^. The specific endoscopic features of CMV esophagitis are solitary, large, deep, punch-out, or demarcated serpiginous ulcers^2,4,8^. However, the endoscopic features of CMV esophagitis are variable. CMV esophagitis commonly involves multiple ulcers varying in size in the middle to distal esophagus. The depths of CMV esophageal ulcers are more commonly shallow or intermediate than deep and healed-up^9^. The endoscopic features between HSV and CMV esophagitis significantly overlap^1,8^. Therefore, the differential diagnosis between HSV and CMV esophagitis using endoscopic features can sometimes be confusing.

Recently, many studies have reported impressive performances of artificial intelligence (AI) systems for medical imaging^10,11^. Using a large dataset, an AI system can compensate for the experience of experts and identify microstructures and quantitative pixel-level features which are undetectable by the human eye ^12^. In gastrointestinal (GI) endoscopy, several studies have shown favorable performance for detecting and classifying GI neoplasms^13^. Also, AI algorithms for benign, chronic inflammatory disease with diffuse involvement, such as *Helicobacter pylori* gastritis, have reported high accuracy in diagnosis using endoscopic images^14,15^. Nevertheless, a shortcoming of deep learning is that a large amount of data is needed to minimize overfitting and improve learning^16^. Therefore, image feature-based classifiers could be a better classification strategy for small datasets^17,18^.

In this study, we aimed to develop a machine-learning-based AI system for differential diagnosis between HSV and CMV esophagitis using endoscopic images. The classification task can be greatly affected by the extraction and classification of different features. To capture better endoscopic features of HSV and CMV esophagitis, we manually annotated the regions of interest (ROIs). Subsequently, the image features were extracted from the annotated ROIs of the endoscopic color images, which were represented by the hue–saturation–brightness (HSB) color model. After channel-wise feature filtering based on each channel of color model, the final features were selected by a least absolute shrinkage and selection operation (LASSO), and then machine learning classifiers were trained. In order to achieve robust performance, ROI-based classifiers were designed instead of image-based classifiers, and image-based and patient-based accuracies were then obtained by ensembling the results of the ROIs.

## Results

### Baseline and endoscopic characteristics of patients

The clinical and endoscopic characteristics of the 150 patients are summarized in Table 1. Out of 150 patients, 87 were diagnosed with HSV esophagitis and 63 with CMV esophagitis. The median age was 61 years (interquartile range 51–70 years) and 119 patients (79.3%) were immunocompromised. There were no significant differences in age, sex, or comorbidities except for solid organ transplantation. Solid organ transplantation was significantly more common in patients with CMV esophagitis than in those with HSV esophagitis (36.5% vs. 12.6%, *p* < 0.001).

**Table 1:** Baseline and endoscopic characteristics of 150 patients with HSV and CMV esophagitis.

	HSV (n = 87)	CMV (n = 63)	*p* value
**Age (median, IQR) (years)**	59 (24–84)	61 (24–79)	0.214
**Sex (male:female)**	61:26	23:40	0.393
**Comorbidity**			
Malignancies	34 (39.1)	22 (34.9)	0.603
Solid tumor	27 (31.0)	19 (30.2)	0.909
Hematologic malignancy	7 (8.0)	3 (4.8)	0.521
Immunosuppressive therapy			
Transplantation	14 (16.1)	24 (38.1)	0.002
Solid organ	11 (12.6)	23 (36.5)	0.001
Hematopoietic stem cell	3 (3.4)	1 (1.6)	0.639
Rheumatologic disease	6 (6.9)	2 (3.2)	0.469
HIV infection	0	2 (3.2)	0.175
Diabetes mellitus	14 (16.1)	17 (27.0)	0.139
Chronic kidney disease	4 (4.6)	3 (4.8)	1.000
Liver cirrhosis	1 (1.1)	2 (3.2)	0.572
Steroid user*	2 (2.3)	0	0.510
Corrosive esophagitis	1 (1.1)	0	1.000
No underlying disease	4 (4.6)	0	0.139
**Distribution**			0.111
Proximal esophagus	5 (5.7)	3 (4.8)	1.000
Middle esophagus	10 (11.5)	12 (46.0)	0.197
Distal esophagus	25 (28.7)	23 (23.8)	0.314
Two or more segments	47 (54.0)	25 (39.7)	0.083
**Initial diagnosis of endoscopist**			
HSV esophagitis	50 (57.5)	5 (7.9)	
CMV esophagitis	1 (1.1)	29 (46.0)	
Indeterminate	12 (13.8)	15 (23.8)	
Esophageal cancer	2 (2.3)	3 (4.8)	
Candida esophagitis	11 (12.6)	3 (4.8)	
Reflux esophagitis	5 (5.7)	5 (7.9)	
Non-specific erosion or ulcer	5 (5.7)	2 (3.2)	
Radiation-induced esophagitis	1 (1.1)	1 (1.6)	

Data are the number of patients (%) unless otherwise noted.

*IQR*, interquartile range, *HSV* herpes simplex virus, *CMV* cytomegalovirus, *HIV* human immunodeficiency virus.

*Patients treated with steroid for asthma or interstitial lung disease.

The distribution of HSV and CMV esophagitis commonly involved two or more segments of the esophagus. In cases of esophagitis involving two or more segments, 53.1% (25/47) of patients with HSV esophagitis and 52% (13/25) of patients with CMV esophagitis had involvement of the middle to distal esophagus. Therefore, the middle and/or distal esophagus were the most involved regions in HSV and CMV esophagitis (94.3% and 95.2%, respectively).

The initial endoscopic diagnosis based on the morphologic findings at the time of endoscopy varied among HSV or CMV esophagitis, reflux esophagitis, and esophageal cancer. Compared with the definite diagnosis, only 57.5% (50/87) of HSV esophagitis cases and 46% (29/63) of CMV esophagitis cases were initially diagnosed by endoscopic features at the time of endoscopy. The overall diagnostic accuracy of endoscopists was 52.7% (79/150). There was no significant difference between the diagnostic accuracy of endoscopists (*p* = 0.166) for HSV and CMV esophagitis.

### Development and performance of the AI system for differential diagnosis between HSV and CMV esophagitis

The classifiers were trained using five repeated five-fold cross-validations in a stratified manner over patients, and they evaluated per-ROI, per-image, and per-patient performances using datasets divided according to the patients. We obtained the image-based and patient-based accuracies from the designed ROI-based classifier through an averaged probability. The probabilities of all ROIs in one image or one patient were averaged and considered the representative probability of the image or patient, respectively. Using these representative probabilities, final diagnoses were made. Classifiers based on an HSB color model surpassed classifiers based on an RGB color model in all classification metrics (Tables 2, 3 and 4). In the case of the HSB color model with superior performance, per-patient accuracies were 100% in all models; therefore, it was difficult to compare the performances between models. For performance comparison between models, the per-image accuracies in the HSB color model were summarized as follows. Logistic regression with LASSO showed the best performance; the sensitivity, specificity, PPV, NPV, accuracy, and AUC were 100%, 100%, 100%, 100%, 100%, and 1.0, respectively. It is recommended to perform random forest classification with LASSO; the sensitivity, specificity, PPV, NPV, accuracy, and AUC were 99.8%, 99.4%, 99.1%, 99.8%, 99.6%, and 1.0, respectively, using LASSO. Previous history of transplantation was included in the features as a clinical factor, and the lower the performance of classifiers, the greater the effect of including this clinical factor. As a result of evaluating the differences in diagnostic performance between models using the Wilcoxon signed-rank test^19^, significant differences (*p* value < 0.05) were observed among three models (logistic regression with LASSO, random forest with LASSO, and random forest) in the case of the HSB color model, but no significant difference was noted in the case of the RGB color model (Supplementary Table S5).

**Table 2:** Diagnostic performance of logistic regression with LASSO for discriminating cytomegalovirus esophagitis from herpes simplex virus esophagitis.

Logistic regression with LASSO	Sen. (%)	Spec. (%)	PPV (%)	NPV (%)	Acc (%)	AUC
**HSB color model**						
Without clinical factor* (per-ROI/per-image/per-patient)	100 (0.0)	100 (0.0)	100 (0.0)	100 (0.0)	100 (0.0)	1.0 (0.0)
	100 (0.0)	100 (0.0)	100 (0.0)	100 (0.0)	100 (0.0)	1.0 (0.0)
	100 (0.0)	100 (0.0)	100 (0.0)	100 (0.0)	100 (0.0)	1.0 (0.0)
With clinical factor* (per-ROI/per-image/per-patient)	100 (0.0)	100 (0.0)	100 (0.0)	100 (0.0)	100 (0.0)	1.0 (0.0)
	100 (0.0)	100 (0.0)	100 (0.0)	100 (0.0)	100 (0.0)	1.0 (0.0)
	100 (0.0)	100 (0.0)	100 (0.0)	100 (0.0)	100 (0.0)	1.0 (0.0)
**RGB color model**						
Without clinical factor* (per-ROI/per-image/per-patient)	71.6 (17.1)	65.8 (14.7)	42.0 (7.5)	88.3 (5.2)	67.9 (8.8)	0.737 (0.073)
	76.1 (15.1)	58.6 (18.8)	55.6 (11.3)	81.0 (8.2)	65.0 (8.4)	0.709 (0.080)
	83.2 (16.1)	61.3 (16.2)	62.4 (8.6)	86.3 (10.8)	70.5 (6.9)	0.710 (0.091)
With clinical factor* (per-ROI/per-image/per-patient)	73.6 (15.0)	68.9 (11.0)	43.7 (8.0)	89.2 (5.7)	70.3 (7.0)	0.761 (0.077)
	75.7 (15.3)	65.6 (16.8)	59.8 (10.7)	82.7 (7.6)	69.5 (7.4)	0.746 (0.071)
	79.6 (14.4)	71.0 (16.3)	69.1 (11.4)	84.4 (8.3)	74.7 (7.0)	0.748 (0.079)

Results were obtained per-ROI (top), per-image (center), and per-patient (bottom), and presented as average (standard deviation) of five repeated five-fold cross-validation.

*ROI* region of interest, *HSB* hue–saturation–brightness, *RGB* red–green–blue, *Sen.* sensitivity, *Spec.* specificity, *PPV* positive predictive value, *NPV* negative predictive value, *Acc.* accuracy, *AUC* area under the ROC curve, *ROC* receiver operating characteristic.

*Clinical factor: previous history of transplantation.

**Table 3:** Diagnostic performance of random forest with LASSO for discriminating cytomegalovirus esophagitis from herpes simplex virus esophagitis.

Random forest with LASSO	Sen. (%)	Spec. (%)	PPV (%)	NPV (%)	Acc (%)	AUC
**HSB color model**						
Without clinical factor* (per-ROI/per-image/per-patient)	99.3 (0.8)	98.8 (0.9)	96.5 (2.7)	99.8 (0.3)	99.0 (0.7)	0.999 (0.001)
	99.7 (0.9)	99.7 (0.5)	99.5 (0.7)	99.8 (0.5)	99.7 (0.5)	1.0 (0.001)
	100 (0.0)	100 (0.0)	100 (0.0)	100 (0.0)	100 (0.0)	1.0 (0.0)
With clinical factor* (per-ROI/per-image/per-patient)	99.2 (0.8)	98.8 (0.7)	96.2 (2.8)	99.7 (0.3)	98.9 (0.7)	0.999 (0.001)
	99.8 (0.6)	99.4 (0.8)	99.1 (1.2)	99.8 (0.4)	99.6 (0.6)	1.0 (0.001)
	100 (0.0)	100 (0.0)	100 (0.0)	100 (0.0)	100 (0.0)	1.0 (0.0)
**RGB color model**						
Without clinical factor* (per-ROI/per-image/per-patient)	72.0 (13.0)	67.1 (12.9)	42.6 (9.8)	88.4 (5.1)	68.5 (7.8)	0.745 (0.066)
	71.8 (14.1)	68.5 (13.6)	59.9 (8.7)	81.0 (7.0)	70.0 (6.0)	0.737 (0.073)
	78.0 (16.6)	74.6 (18.0)	72.6 (13.3)	84.6 (9.8)	76.1 (8.0)	0.752 (0.093)
With clinical factor* (per-ROI/per-image/per-patient)	65.2 (16.8)	73.4 (12.4)	45.3 (11.8)	87.1 (6.1)	71.2 (7.0)	0.741 (0.082)
	68.9 (13.8)	72.7 (16.3)	63.9 (12.5)	79.9 (6.4)	71.2 (6.9)	0.749 (0.076)
	77.1 (16.1)	75.0 (16.3)	72.6 (13.7)	83.9 (8.7)	76.1 (7.1)	0.769 (0.075)

Results were obtained per-ROI (top), per-image (center), and per-patient (bottom), and presented as average (standard deviation) of five repeated five-fold cross-validation.

*ROI* region of interest, *HSB* hue–saturation–brightness, *RGB* red–green–blue, *Sen.* sensitivity, *Spec.* specificity, *PPV* positive predictive value, *NPV* negative predictive value, *Acc.* accuracy, *AUC* area under the ROC curve, *ROC* receiver operating characteristic.

*Clinical factor: previous history of transplantation.

**Table 4:** Diagnostic performance of random forest for discriminating cytomegalovirus esophagitis from herpes simplex virus esophagitis.

Random forest	Sen. (%)	Spec. (%)	PPV (%)	NPV (%)	Acc (%)	AUC
**HSB color model**						
Without clinical factor* (per-ROI/per-image/per-patient)	98.7 (0. 9)	98.3 (1.3)	94.9 (4.3)	99.6 (0.3)	98.4 (1.1)	0.998 (0.002)
	99.5 (0.8)	99.0 (1.2)	98.4 (2.1)	99.7 (0.5)	99.2 (0.9)	0.999 (0.001)
	100 (0.0)	100 (0.0)	100 (0.0)	100 (0.0)	100 (0.0)	1.0 (0.0)
With clinical factor* (per-ROI/per-image/per-patient)	98.3 (1.7)	98.2 (0.9)	94.3 (3. 6)	99.5 (0.5)	98.3 (0.9)	0.998 (0.002)
	99.4 (1.1)	99.2 (1.0)	98.7 (1.6)	99.6 (0.6)	99.3 (0.9)	0.999 (0.001)
	100 (0.0)	100 (0.0)	100 (0.0)	100 (0.0)	100 (0.0)	1.0 (0.0)
**RGB color model**						
Without clinical factor* (per-ROI/per-image/per-patient)	67.4 (11.8)	74.0 (10.9)	46.8 (11.2)	87.5 (5.3)	72.2 (7.4)	0.752 (0.067)
	65.2 (15.0)	74.3 (11.2)	62.6 (7.9)	78.2 (6.4)	70.9 (4.4)	0.734 (0.063)
	70.8 (11.3)	79.6 (15.0)	74.4 (13.3)	79.6 (5.0)	75.9 (6.8)	0.754 (0.080)
With clinical factor* (per-ROI/per-image/per-patient)	66.9 (12.1)	74.8 (9.6)	47.3 (10.8)	87.7 (4.8)	73.2 (6.4)	0.752 (0.070)
	68.9 (15.9)	71.5 (11.6)	61.4 (6.8)	79.9 (6.6)	71.0 (4.5)	0.736 (0.064)
	74.1 (13.7)	78.8 (11.6)	73.3 (10.6)	81.8 (6.9)	76.9 (6.3)	0.758 (0.082)

Results were obtained per-ROI (top), per-image (center), and per-patient (bottom), and presented as average (standard deviation) of five repeated five-fold cross-validation.

*ROI* region of interest, *HSB* hue–saturation–brightness, *RGB* red–green–blue, *Sen.* sensitivity, *Spec.* specificity, *PPV* positive predictive value, *NPV* negative predictive value, *Acc.* accuracy, *AUC* area under the ROC curve, *ROC* receiver operating characteristic.

*Clinical factor: previous history of transplantation.

## Discussion

We established an AI system with good performance based on endoscopic images for differential diagnosis between HSV and CMV esophagitis. The AI system was trained and validated using 1082 endoscopic images from 150 patients. Our machine-learning-based AI system, which used logistic regression with LASSO for discriminating CMV esophagitis from HSV esophagitis, showed a sensitivity, specificity, PPV, NPV, accuracy, and AUC of 100%, 100%, 100%, 100%, 100%, and 1.0, respectively. To the best of our knowledge, this is the first AI system using endoscopic images with a clinical factor for differential diagnosis between HSV and CMV esophagitis.

Although histopathology with specific IHC stains is the gold standard for the diagnosis of HSV and CMV esophagitis, endoscopic features are important for empirical treatment prior to histopathologic diagnosis because tissue-based diagnostic evaluation takes several days^1^. It is very important to start proper treatment as quickly as possible and within a few days, especially for immunocompromised patients. Several studies have reported endoscopic features for HSV or CMV esophagitis^2,7,9,20^. However, these features significantly overlap in site involvement as they both feature mainly multiple small-sized and shallow ulcers^1^. In our study, the overall diagnostic accuracy of endoscopic features was only 52.7%, which means that nearly 50% of patients may receive erroneous empirical treatment until histopathology results are obtained. The differential diagnosis between HSV and CMV esophagitis based on endoscopic features will be the most important prognostic parameter for immunocompromised patients, in whom rapid treatment can determine prognosis.

Recently, our group investigated the implications of using endoscopic findings for the diagnosis of HSV and CMV esophagitis^21^. The average diagnostic accuracy of eight highly experienced endoscopists was 74.3%, and about a quarter of the patients diagnosed as HSV or CMV esophagitis based on endoscopic features were misdiagnosed regardless of the endoscopists’ expertise. Therefore, we developed a predictive model based on the categorization of endoscopic features and history of transplantation with a high accuracy (92.6%) in discriminating CMV esophagitis from HSV esophagitis. Training through categorizing endoscopic features can help endoscopists make accurate diagnoses, but sufficient training is difficult because of the rarity of CMV and HSV esophagitis. Machine learning approaches using retrospective data can overcome dependency on experience and the rarity of the disease.

The classification task can be greatly affected by different feature extraction and classification methods. To capture better endoscopic features of HSV and CMV esophagitis, we manually annotated ROIs with the assistance of an expert endoscopist and then extracted image features using an HSB color model. The accuracy of the HSB color model was significantly better than that of the RGB color model, because the HSB color model is designed to approximate the way humans perceive and interpret color and could be a device-independent color representation format^22^. The robust performance was achieved by averaging the results of the ROI-based classifiers. In our study, the diagnostic accuracy of the developed classifier (logistic regression with LASSO) in discriminating CMV esophagitis from HSV esophagitis was 100%, which is better than that of the initial diagnoses by endoscopists (100% vs. 52.7%) as well as that of experienced endoscopists (100% vs. 74.3%) reported previously^21^. The developed AI system has potential for clinical application in differential diagnosis between HSV and CMV esophagitis.

Some methodological limitations of this study should be noted. First of all, our study design was retrospective in nature and had a small sample size. However, viral esophagitis is rare in immunocompetent patients and is an opportunistic disease in immunocompromised patients. Additionally, to the best of our knowledge, this study is the largest study of HSV and CMV esophagitis, respectively. The development of an AI system using images is needed for a large dataset of high-quality images. Therefore, considering the rarity of HSV and CMV esophagitis, our study enrolled the largest number of HSV and CMV esophagitis cases and developed an AI system for differential diagnosis between HSV and CMV esophagitis. Second, we did not perform comparisons between endoscopists and our AI system for validation. We previously reported differential diagnosis between HSV and CMV esophagitis using categorization of endoscopic features^21^. In that study, the diagnostic accuracy of endoscopists in randomly selected cases of esophagitis was 74.3% in the experienced group and 74.7% in the less experienced group. A highly experienced endoscopist categorized the endoscopic features and the diagnostic accuracy improved to 92.6%. Therefore, the categorization of endoscopic features is dependent on the experience of endoscopists. Our AI system can compensate for expert experience and can support less experienced endoscopists. Finally, ROI annotation is required for the developed AI system. We have already assigned ROIs with the help of an expert, and this dataset can be used for training an AI system for ROI annotation, enabling an end-to-end system.

In conclusion, our machine-learning-based AI system using logistic regression with LASSO for differential diagnosis between HSV and CMV esophagitis showed high accuracy. The improvement of the diagnostic accuracy of clinicians through this AI system will contribute to improving the prognosis of patients by providing rapid treatment based on a quick prediction.

## Materials and methods

### Patients and date collection

We retrospectively reviewed the medical records and endoscopic images of all patients diagnosed with HSV or CMV esophagitis between April 2008 and December 2016 at Asan Medical Center (Seoul, Korea). The diagnosis of HSV or CMV esophagitis was confirmed with clinical symptoms, endoscopic findings, and histopathologic review with IHC and/or PCR. Patients were excluded according to the following criteria: co-infection with HSV and CMV, final pathologic diagnosis of malignancy, recurrent infection, or missing information on endoscopic findings. The institutional review board of Asan Medical Center approved the study (IRB No. 2020-0495). Due to the retrospective study design, written informed consent was not obtained from participants. The IRB of our institution waived the need for informed consent based on the non-invasive and anonymized nature of this study. This study was conducted in accordance with institutional ethical guidelines and the Declaration of Helsinki.

### Lesion segmentation and feature extraction

**Figure 1:**
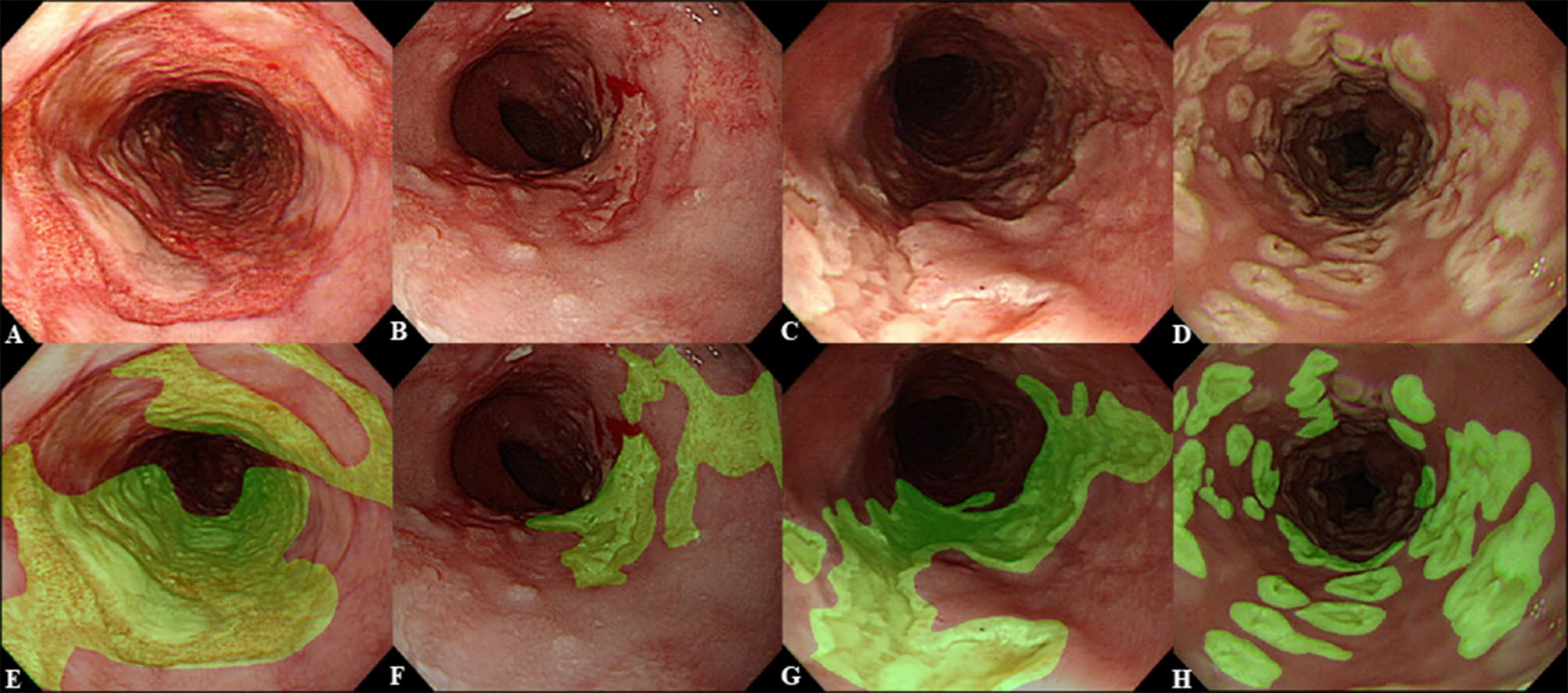
Regions of interest (ROIs) of cytomegalovirus esophagitis and herpes simplex virus esophagitis. (**A**,**B**) Cytomegalovirus esophagitis cases. (**C**,**D**) Herpes simplex virus esophagitis cases. (**E**–**H**) Manually annotated ROIs of esophagitis lesions.

In order to extract imaging features to differentiate between the two types of esophagitis, one board-certified expert (more than 15 years of experience in endoscopy) reviewed the quality of the collected endoscopic images and manually annotated the regions of interest (ROIs). Cases of shaky images or lesions far away from the endoscope light source were excluded because the shapes of the lesions were not clearly visible. ROIs were drawn as close to the margins of the lesions as possible so as to not include the normal esophageal mucosa (Fig. 1).

The hue–saturation–brightness (HSB) color model was employed to extract image features from endoscopic color images. In color image processing, there are various color models designed for specific purposes, such as red–green–blue (RGB), cyan–magenta–yellow–black (CMYK), and HSB. The HSB color model, which was designed to approximate the way humans perceive and interpret color, is often used in computer vision for feature detection or image segmentation since it is a device-independent color representation format^22^. Our esophagitis classifier was compared with one based on the RGB color model, which is the most widely used. Since the characteristics of each ROI in the image are expected to be different, ROI-based classifiers were designed instead of image-based classifiers, and then image-based accuracy was obtained by averaging the results of the ROIs. We collected 1082 endoscopic images from 150 patients, obtaining a total of 3444 ROIs (HSV: 87 patients, 666 endoscopic images, 2628 ROIs; CMV: 63 patients, 416 endoscopic images, 816 ROIs).

There were 520 image features extracted from each channel of the HSB and RGB color models, resulting in a total of 1,560 image features extracted from each ROI, including first-order (N = 17), texture (N = 87) and wavelet analyses (N = 416) (Supplementary Appendix I). The first-order features were derived from intensity histograms using first-order statistics, including intensity range, energy, entropy, kurtosis/skewness, maximum/minimum, mean, median, uniformity, and variance. Texture features were obtained with a gray-level co-occurrence matrix (GLCM) and a gray-level run length matrix (GLRLM) in four directions in two-dimensional (2D) space^23^; GLCM texture features were computed for varying distances of 1, 2, and 3 pixels in four directions. The wavelet transformation was applied with a single-level directional discrete wavelet transformation of high-pass and low-pass filters^24^. In total, four wavelet-decomposition images were generated from each ROI: LL, LH, HL, and HH images, where ‘L’ means ‘low-pass filter’ and ‘H’ means ‘high-pass filter.’ Then, the first-order and texture features were applied to the wavelet-transformed images, yielding 416 wavelet features (17 first-order and 87 texture features per wavelet-transformed image). All image features were standardized by z-transformation before applying classification metrics.

### Classification metrics

Effective feature selection is a crucial step because image features are multiple collinear and correlated predictors that could produce unstable estimates and might overfit predictions. The feature selection methods can be divided by how they are coupled to the classification or learning algorithms as follows: (1) filter method, (2) wrapper method, (3) embedded method^25^. Filter methods reduce the number of features independently. Wrapper methods wrap the feature selection around the classification method and use the prediction accuracy of the model to iteratively select or eliminate a set of features. In embedded methods, the feature selection process is an integral part of the classification model. We made feature selection more efficient by combining the filter method (i.e., feature filtering using univariate feature selection) and the embedded method (i.e., LASSO). First, we filtered the extracted features using univariate feature selection in terms of each channel of the HSB and RGB color models. Based on the *p* value (< 0.05) of ANOVA tests, 124 features of HSB color models were filtered out, and the remaining features included 478 H-channel features, 481 S-channel features, and 477 B-channel features. For the RGB color model, 420 features were filtered out, and the remaining features included 341 R-channel features, 410 G-channel features, and 389 B-channel features. After channel-wise feature filtering, the remaining features were combined according to color model (HSB color model: 1436 features, RGB color model: 1140 features). A LASSO was then employed for feature selection of combined features. A total of 25 LASSOs were performed by five repeated five-fold cross-validations, and 11–18 features and 11–20 features were selected from the HSB and RGB color models, respectively (Supplementary Appendix II). Using selected image features, two different machine learning classifiers were trained: logistic regression and random forest. The random forest is a classifier that derives and ensembles several decision tree classifiers on various sub-samples of the dataset to improve the predictive accuracy and control overfitting. In other words, random forest does not require additional feature selection. However, we tried to improve the performance of random forest by combining LASSO since our dataset has many features compared with the number of datasets. While performing five repeated five-fold cross-validations, the hyperparameters of logistic regression and random forest were obtained by nested cross-validation in each fold. To maximize the probabilities of correct decisions, we found an optimal cutoff value using the true-positive and false-positive rates forming the receiver operating characteristic (ROC) curve^26^. Univariate feature selection, LASSO, logistic regression, and random forest classification were implemented using the Scikit-learn package (https://github.com/scikit-learn/scikit-learn)^27^.

### Statistics

Categorical data were analyzed using the chi-squared test or Fisher’s exact test as appropriate. Numerical data were analyzed using Student’s t-test. Sensitivity, specificity, positive predictive value (PPV), negative predictive value (NPV), accuracy, and area under the curve (AUC) were calculated by standard definitions to evaluate the performance of the developed AI system. To evaluate the differences in performance between models, we performed the Wilcoxon signed-rank test^19^. All statistical analyses were performed using SPSS Statistics for Windows, version 18.0 (IBM; Armonk, NY). *p* values < 0.05 were considered statistically significant.

## Supplementary information

Supplementary Information.
