# Supplementary material for: Machine learning approach for differentiating cytomegalovirus esophagitis from herpes simplex virus esophagitis
Source: Sci Rep. 2021 Feb 11;11:3672. doi: 10.1038/s41598-020-78556-z (PMC7878749; doi:10.1038/s41598-020-78556-z)
Supplement: Supplementary file 1 — Supplementary Information. [file 41598_2020_78556_MOESM1_ESM.docx]

**Machine learning approach for differentiating cytomegalovirus esophagitis from herpes simplex virus esophagitis**

**Jung Su Lee**^1, 2,+^, MD, **Jihye Yun**^3,+^, PhD, Sungwon Ham^4^, MS, Hyunjung Park^5^, BS, Hyunsu Lee^6^, MD, PhD, Jeongseok Kim^7^, MD, Jeong-Sik Byeon^1^, MD, PhD, Hwoon-Yong Jung^1^, MD, PhD, AGAF, FASGE, **Namkug Kim**^3, 4,*^, PhD, and **Do Hoon Kim**^1,*^, MD, PhD

^1^Department of Gastroenterology, Asan Medical Center, University of Ulsan College of Medicine, Seoul, Republic of Korea

^2^Department of Gastroenterology, Ilsan Paik Hospital, Inje University College of Medicine, Goyang, Republic of Korea

^3^Department of Radiology, Asan Medical Center, University of Ulsan College of Medicine, Seoul, Republic of Korea

^4^Department of Convergence Medicine, Asan Medical Institute of Convergence Science and Technology, Asan Medical Center, University of Ulsan College of Medicine, Seoul, Republic of Korea

^5^Asan Institute for Life Sciences, Asan Medical Center, Seoul, Republic of Korea

^6^Department of Anatomy, Keimyung University School of Medicine, Daegu, Republic of Korea

^7^Department of Internal Medicine, Keimyung University School of Medicine, Daegu, Republic of Korea

^+^These two authors contributed equally to this work.

^*^These two authors contributed equally as the corresponding authors.

Supplementary Appendix I

The calculated image features were divided into three groups: 17 first-order statistics, 87 texture features, and 416 wavelet features. The wavelet transformation was applied to the original images, obtaining four wavelet-decomposition images. Then, the first-order and texture features were applied to the wavelet-transformed images, yielding 416 wavelet features.

**First-order statistics**

First-order statistics were calculated from the histogram of pixel intensities, which represents the distribution of gray values within an image. Let $P$ denote the first-order histogram of a region of interest $I\left( x, y \right)$ and $P\left( i \right)$ the number of pixels with gray-level $i$. The number of gray-level bins set for $P$ is represented as $N_{g}$. The $i$-th probability vector of the first-order histogram is then defined as follows:

$$p\left( i \right)=\frac{P\left( i \right)}{\sum_{i=1}^{N_{g}} P\left( i \right)}$$

Let $I_{ROI}$ and $I$ denote the intensity values of all pixels within $I\left( x, y \right)$ with $N$ pixels and the whole image. The mean and center gray values within $I\left( x, y \right)$ are $\bar{I}$ and $C$, respectively. Table S1 summarizes the employed first-order statistics of image features.

**Texture features**

Although first-order features provide information on the gray-level distribution of the region of interest, they do not describe information related to the relative positions of the various gray-levels of the region of interest (ROI). The methods most often used for texture analysis are the gray-level co-occurrence matrix (GLCM) and the gray-level run length matrix (GLRLM). In GLCM, various textural features are extracted, and GLRLM characterizes coarse textures as having many pixels in a constant gray-level run and fine textures as having few pixels in such a run. Both GLCM and GLRLM are matrix-based features and are constructed from 2D analysis of an ROI with 4-connectivity.

**GLCM features**

Let $G$ denote the GLCM of a quantized image $I(x,y)$ and let $G_{\alpha,\delta}(i,j)$ represent the number of times that pixels of gray-level $i$ were neighbors with pixels of gray-level $j$ in $I(x, y)$ in 1 of 4 directions of $\alpha$ and at a distance $\delta=1,2,3$. GLCM is the size of $N_{g}\times N_{g}$ where $N_{g}$ describes a pre-defined number of quantized gray-level sets in $I(x,y)$. For each direction $\alpha$ and distance $\delta$, the normalized GLCM is obtained as follows:

$g_{\alpha,\delta}\left( i,j \right)=g\left( i,j \right)=\frac{G\left( i,j \right)}{\sum_{i=1}^{N_{g}} \sum_{j=1}^{N_{g}} G\left( i,j \right)}$.

Frequently used feature quantities for each direction and distance are also defined as follows:

$\mu$ is the mean of $g\left( i,j \right)$,

$\sigma$ is the standard deviation of $g\left( i,j \right)$,

$g_{x}\left( i \right)$ is the marginal row probability of $g\left( i,j \right)$: $g_{x}\left( i \right)=\sum_{j}^{N_{g}} g\left( i,j \right)$,

$\mu_{x}$ is the mean of $g_{x}\left( i \right)$,

$\sigma_{x}$ is the standard deviation of $g_{x}\left( i \right)$,

$HX=-\sum_{i=1}^{N_{g}} g_{x}\left( i \right) \log_{2} \left( g_{x}\left( i \right)+\varepsilon\right)$ is the entropy of $g_{x}\left( i \right)$,

$HXY=-\sum_{i=1}^{N_{g}} \sum_{j=1}^{N_{g}} g\left( i,j \right) \log_{2} \left( g\left( i,j \right)+\varepsilon\right)$ is the entropy of $g\left( i,j \right)$,

$HXY1=-\sum_{i=1}^{N_{g}} \sum_{j=1}^{N_{g}} g\left( i,j \right) \log_{2} \left( g_{x}\left( i \right)g_{y}\left( j \right)+\varepsilon\right)$,

$HXY1=-\sum_{i=1}^{N_{g}} \sum_{j=1}^{N_{g}} g_{x}\left( i \right)g_{y}\left( j \right) \log_{2} \left( g_{x}\left( i \right)g_{y}\left( j \right)+\varepsilon\right)$.

Table S2 summarizes the GLCM image features used.

**GLRLM features**

Let $Q$ denote the GLRLM of a quantized image $I(x,y)$ and $Q(i,j)$ represent the number of runs of gray-level $i$ with $j$ consecutive pixels in one of the four directions of $\alpha$. GLRLM is the size of $N_{g}\times N_{l}$ where $N_{g}$ describes the pre-defined number and $N_{l}$ represents the length of the longest run of quantized gray-level sets in $I(x,y)$. $N_{p}$ is the number of pixels in $I(x,y)$. Table S3 summarizes the GLRLM image features used.

Supplementary Appendix II

Table 4S summarizes the selected features for ROI-based classifiers according to HSB and RGB models. Because extracted features may be noisy or highly correlated with each other, feature selection is required to increase accuracy of prediction and minimize computational costs[6]. We filtered the extracted features using univariate feature selection in terms of each channel of HSB and RGB color models, obtaining 1,436 features for HSB color model and 1,140 features for RGB color model. The final features were selected using a least absolute shrinkage and selection operation (LASSO) with $\alpha=0.05$. A total of 25 LASSOs were performed with five repeated five-fold cross-validations, and 11~18 features and 11~20 features were selected from the HSB and RGB color models, respectively. We summarized the selection frequency along with the top 15 features to be able to analyze the importance of the features.

Supplementary Tables

**Table S1.** First-order statistics

| Number of pixels | $\left\vert I_{ROI} \right\vert$ | Sum of intensity | $\sum_{i}^{N} \boldsymbol{I}_{ROI}(i)$ |
| --- | --- | --- | --- |
| Range | $\text{max}\left( I_{ROI} \right)-\text{min}\left( I_{ROI} \right)$ | Energy | $N^{2}\sum_{i}^{N_{g}} {p\left( i \right)}^{2}$ |
| Covered image intensity range | $\frac{\max I_{ROI}-\min I_{ROI}}{\left( \max I-\min I \right)}$ | Entropy | $-\sum_{i}^{N_{g}} \left[ p\left( i \right)*\log_{2} \left( p\left( i \right) \right) \right]$ |
| Maximum intensity value | $\max\left( I_{ROI} \right)$ | Kurtosis | $\frac{\sum_{i}^{N_{g}} \left[ p\left( i \right)*\left( \boldsymbol{C}\left( i \right)- \overline{I} \right)^{4} \right]}{\left( \sum_{i}^{N_{g}} \left[ p\left( i \right)*\left( \boldsymbol{C}\left( i \right)-\overline{I} \right) \right] \right)^{2}}$ |
| Mean intensity value | $\frac{1}{N}\sum_{i}^{N} \boldsymbol{I}_{ROI}(i)$ | Skewness | $\frac{\sum_{i}^{N_{g}} \left[ p\left( i \right)*\left( \boldsymbol{C}\left( i \right)- \overline{I} \right)^{3} \right]}{\left( \sum_{i}^{N_{g}} \left[ p\left( i \right)*\left( \boldsymbol{C}\left( i \right)- \overline{I} \right)^{3} \right] \right)^{\frac{3}{2}}}$ |
| Median intensity value | $\mathrm{med} \left( \boldsymbol{I}_{ROI} \right)$ | Root mean square | $\sum_{i}^{N_{g}} \left[ p\left( i \right)*\boldsymbol{C}\left( i \right)^{2} \right]$ |
| Minimum intensity value | $\min\left( \boldsymbol{I}_{ROI} \right)$ | Variance | $\frac{1}{N-1}\sum_{i}^{N} \left( \boldsymbol{I}_{ROI}\left( i \right)- \overline{I} \right)^{2}$ |
| Mean absolute deviation | $\sum_{i}^{N_{g}} \left[ p\left( i \right)*(\boldsymbol{C}\left( i \right)- \overline{I}) \right]$ | Standard deviation | $\sqrt{\frac{1}{N-1}\sum_{i}^{N} \left( \boldsymbol{I}_{ROI}\left( i \right)- \overline{I} \right)^{2}}$ |
| Uniformity | $\sum_{i}^{N_{g}} p\left( i \right)^{2}$ |  | |

**Table S2.** GLCM features

| Autocorrelation | $\sum_{i}^{N_{g}} \sum_{j}^{N_{g}} i*j*g(i,j)$ | Inverse difference | $\sum_{i}^{N_{g}} \sum_{j}^{N_{g}} \frac{g\left( i,j \right)}{1+\left\vert i-j \right\vert}$ |
| --- | --- | --- | --- |
| Cluster prominence | $\sum_{i}^{N_{g}} \sum_{j}^{N_{g}} \left( i+j-2\mu\right)^{4}*g\left( i,j \right)$ | Inverse difference normalized | $\frac{1}{N_{g}}\sum_{i}^{N_{g}} \sum_{j}^{N_{g}} \frac{g\left( i,j \right)}{1+\left\vert i-j \right\vert}$ |
| Cluster shade | $\sum_{i}^{N_{g}} \sum_{j}^{N_{g}} \left( i+j-2\mu\right)^{3}*g\left( i,j \right)$ | Inverse difference  moment | $\sum_{i}^{N_{g}} \sum_{j}^{N_{g}} \frac{g\left( i,j \right)}{1+\left( i-j \right)^{2}}$ |
| Cluster tendency | $\sum_{i}^{N_{g}} \sum_{j}^{N_{g}} \left( i+j-2\mu\right)^{2}*g\left( i,j \right)$ | Inverse difference  moment normalized | $\frac{1}{{N_{g}}^{2}}\sum_{i}^{N_{g}} \sum_{j}^{N_{g}} \frac{g\left( i,j \right)}{1+\left( i-j \right)^{2}}$ |
| Contrast | $\sum_{i}^{N_{g}} \sum_{j}^{N_{g}} \left( i-j \right)^{2}*g\left( i,j \right)$ | Inverse variance | $\sum_{i}^{N_{g}} \sum_{j}^{N_{g}} \frac{g\left( i,j \right)}{\left( i-j \right)^{2}}$ |
| Correlation | $\frac{1}{\sigma}\sum_{i}^{N_{g}} \sum_{j}^{N_{g}} \left( i-\mu\right)\left( j-\mu\right)*g\left( i,j \right)$ | Maximum probability | $max\left( g\left( i,j \right) \right)$ |
| Difference average | $\sum_{k}^{N_{g}} k*g_{x-y}\left( k \right)$ | Sum average | $\sum_{k}^{2N_{g}} i*g_{x+y}\left( k \right)$ |
| Difference entropy | $-\sum_{k}^{N_{g}} g_{x-y}\left( k \right)*{log}_{2}\left( g_{x-y}\left( k \right) \right)$ | Sum entropy | $-\sum_{k}^{2N_{g}} g_{x+y}\left( k \right)*{log}_{2}\left( g_{x+y}\left( k \right) \right)$ |
| Difference variance | $\sum_{k}^{N_{g}} \left( k-\bar{g_{x-y}} \right)^{2}*g_{x-y}\left( k \right)$ | Sum variance | $\sum_{k}^{2N_{g}} \left( k-\bar{g_{x+y}} \right)^{2}*g_{x+y}\left( k \right)$ |
| Dissimilarity | $\sum_{i}^{N_{g}} \sum_{j}^{N_{g}} \left\vert i-j \right\vert*g\left( i,j \right)$ | Variance | $\sum_{i}^{N_{g}} \sum_{j}^{N_{g}} \left( i-\mu\right)^{2}g\left( i,j \right)$ |
| Energy | $\sum_{i}^{N_{g}} \sum_{j}^{N_{g}} {g\left( i,j \right)}^{2}$ | Information measure of  correlation 1 | $\frac{HXY-HXY1}{\max\left\{ HX, HY \right\}}$ |
| Entropy | $-\sum_{i}^{N_{g}} \sum_{j}^{N_{g}} g\left( i,j \right)*{log}_{2}\left( g\left( i,j \right) \right)$ | Information measure of  correlation 2 | $\left( 1-\exp\left[ -2\left( HXY2-HXY \right) \right] \right)^{\frac{1}{2}}$ |
| Harralick correlation | $\frac{1}{\sigma_{x}}\sum_{i}^{N_{g}} \sum_{j}^{N_{g}} \left( i*j*g\left( i,j \right) \right)-\mu_{x}$ |  | |

**Table S3.** GLRLM features

| Number of runs | $N_{run}$ | Low gray-level run emphasis | $\frac{1}{N_{run}}\sum_{i}^{N_{g}} \sum_{j}^{N_{l}} \frac{1}{i^{2}}*Q\left( i,j \right)$ |
| --- | --- | --- | --- |
| Gray-level nonuniformity | $\frac{1}{N_{run}}\sum_{i}^{N_{g}} \left( \sum_{j}^{N_{l}} Q\left( i,j \right) \right)^{2}$ | Run length nonuniformity | $\frac{1}{N_{run}}\sum_{j}^{N_{l}} \left( \sum_{i}^{N_{g}} Q\left( i,j \right) \right)^{2}$ |
| High gray-level run emphasis | $\frac{1}{N_{run}}\sum_{i}^{N_{g}} \sum_{j}^{N_{l}} i^{2}*Q\left( i,j \right)$ | Run percentage | $\frac{N_{run}}{N_{p}}$ |
| Long run emphasis | $\frac{1}{N_{run}}\sum_{i}^{N_{g}} \sum_{j}^{N_{l}} j^{2}*Q\left( i,j \right)$ | Short run emphasis | $\frac{1}{N_{run}}\sum_{i}^{N_{g}} \sum_{j}^{N_{l}} \frac{1}{j^{2}}*Q\left( i,j \right)$ |
| Long run high gray-level emphasis | $\frac{1}{N_{run}}\sum_{i}^{N_{g}} \sum_{j}^{N_{l}} {i^{2}*j}^{2}*Q\left( i,j \right)$ | Short run high gray-level emphasis | $\frac{1}{N_{run}}\sum_{i}^{N_{g}} \sum_{j}^{N_{l}} \frac{i^{2}}{j^{2}}*Q\left( i,j \right)$ |
| Long run low gray-level emphasis | $\frac{1}{N_{run}}\sum_{i}^{N_{g}} \sum_{j}^{N_{l}} \frac{j^{2}}{i^{2}}*Q\left( i,j \right)$ | Short run low gray-level emphasis | $\frac{1}{N_{run}}\sum_{i}^{N_{g}} \sum_{j}^{N_{l}} \frac{1}{i^{2}*j^{2}}*Q\left( i,j \right)$ |

**Table S4.** Selected features for ROI-based classifiers according to the color model

| Color model | Selected features (Top 15) | Selection frequency |
| --- | --- | --- |
| HSB | 1. H / Original / GLCM(1) / Information measure of correlation 1 2. H / Original / GLCM(3) / Information measure of correlation 1 3. H / Original / GLCM(3) / Information measure of correlation 2 4. H / Wavelet(LL) / GLCM(1) / Information measure of correlation 1 5. S / Wavelet(LL) / GLCM(3) / Information measure of correlation 2 6. S / Wavelet(LH) / GLCM(3) / Information measure of correlation 2 7. V / Wavelet(HH) / GLCM(3) / Information measure of correlation 2 8. S / Wavelet(LL) / GLCM(1) / Information measure of correlation 1 9. H / Wavelet(LH) / GLCM(3) / Correlation 10. H / Wavelet(HH) /GLRLM / High gray-level run emphasis 11. H / Wavelet(LH) / GLCM(3) / Information measure of correlation 2 12. H / Wavelet(LH) / GLCM(3) / Information measure of correlation 1 13. S / Wavelet(LL) / GLCM(1) / Information measure of correlation 2 14. S / Wavelet(LL) /GLRLM / Long run high gray-level emphasis 15. V / Wavelet(LH) / GLCM(3) / Information measure of correlation 2 | 25  25  25  25  25  25  25  24  23  23  18  17  11  11  10 |
| RGB | 1. B / Original / GLRLM / Long run low gray-level emphasis 2. R / Original / First-order / Range 3. G / Original / First-order / Range 4. B / Original / GLRLM / Run length nonuniformity 5. B / Wavelet(LL) / GLCM(1) / Information measure of correlation 2 6. B / Wavelet(LL) / GLRLM / Run length nonuniformity 7. B / Original / GLCM(1) / Sum average 8. R / Original / GLCM(3) / Inverse difference moment 9. B / Wavelet(LH) / GLCM(3) / Cluster prominence 10. R / Original / GLCM(3) / Inverse difference moment normalized 11. G / Original / GLRLM / Long run low gray-level emphasis 12. B / Wavelet(LL) / GLCM(1) / Sum entropy 13. B / Wavelet(LL) / GLCM(3) / Energy 14. R / Wavelet(LL) / GLCM(3) / Harralick correlation 15. B / Wavelet(LL) / GLRLM / Short run low gray-level emphasis | 23  21  21  21  21  20  15  13  13  11  11  11  11  10  10 |

Note. Selected image features are presented by “channel of color model /applied image / feature category (first-order, GLCM [distance], or GLRLM) / feature”

**Table S5.** Comparison of performances of the developed models

|  | | Sen.  (%) | Spec.  (%) | PPV  (%) | NPV  (%) | Acc  (%) | AUC |
| --- | --- | --- | --- | --- | --- | --- | --- |
| HSB color model | Without clinical factor*  (model 1vs. model 2 / model 1 vs. model 3 / model 2 vs. model 3) | < 0.05  < 0.05  < 0.05 | < 0.05  < 0.05  < 0.05 | < 0.05  < 0.05  < 0.05 | < 0.05  < 0.05  < 0.05 | < 0.05  < 0.05  < 0.05 | < 0.05  < 0.05  < 0.05 |
|  | With clinical factor*  (model 1vs. model 2 / model 1 vs. model 3 / model 2 vs. model 3) | < 0.05  < 0.05  < 0.05 | < 0.05  < 0.05  < 0.05 | < 0.05  < 0.05  < 0.05 | < 0.05  < 0.05  < 0.05 | < 0.05  < 0.05  < 0.05 | < 0.05  < 0.05  < 0.05 |
| RGB color model | Without clinical factor*  (model 1vs. model 2 / model 1 vs. model 3 / model 2 vs. model 3) | < 0.05  < 0.05  0.67 | 0.08  < 0.05  0.81 | 0.17  < 0.05  0.49 | < 0.05  < 0.05  0.41 | 0.22  < 0.05  0.44 | 0.10  0.54  0.15 |
|  | With clinical factor*  (model 1vs. model 2 / model 1 vs. model 3 / model 2 vs. model 3) | 0.98  0.28  0.16 | 0.53  < 0.05  0.06 | 0.46  < 0.05  < 0.05 | 0.90  0.41  0.40 | 0.46  < 0.05  0.09 | 0.24  0.11  0.25 |

Note. The differences in diagnostic performance between models were evaluated using the Wilcoxon signed-rank test.

*Clinical factor: previous history of transplantation.

Model 1: logistic regression with LASSO; Model 2: random forest with LASSO; Model 3: random forest.
